# Supplementary material for: Nanoparticle-based DNA vaccine protects against SARS-CoV-2 variants in female preclinical models
Source: Nat Commun. 2024 Jan 18;15:590. doi: 10.1038/s41467-024-44830-1 (PMC10796936; doi:10.1038/s41467-024-44830-1)
Supplement: Supplementary file 1 — Supplementary Information [file 41467_2024_44830_MOESM1_ESM.pdf]

Supplementary information

Nanoparticle-based DNA vaccine protects against SARS-CoV-2 variants in female preclinical models

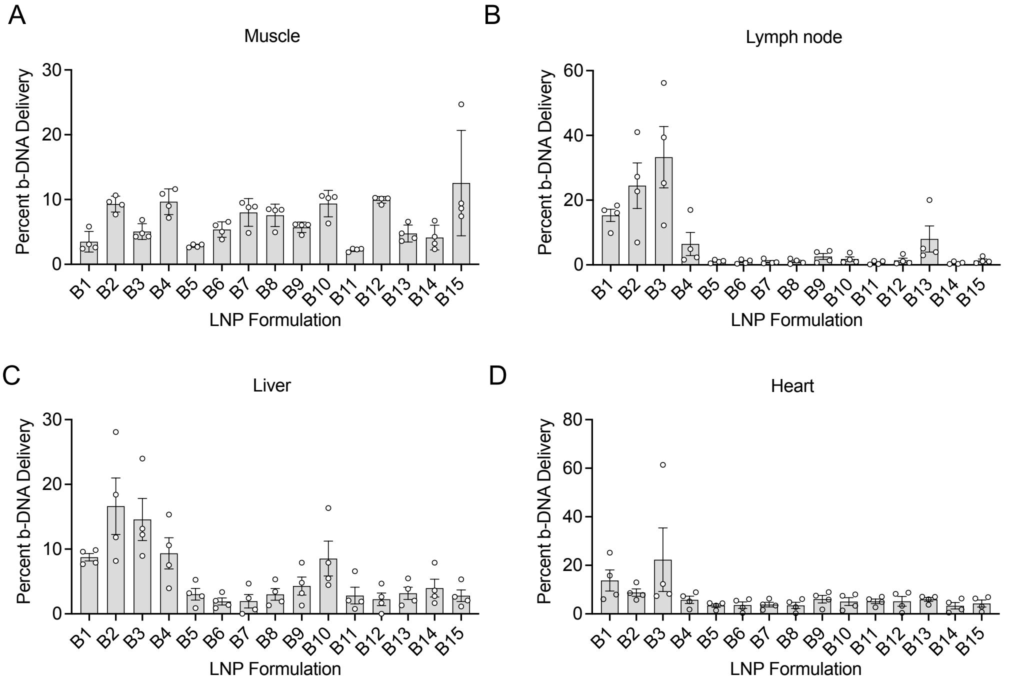

Supplementary Fig. 1: In vivo delivery of LNPs encapsulating barcoded pDNA. The bar graph illustrates the percentage quantification of LNP (B1–B15) delivery in different target tissues 4 hours after intramuscular injection (*n* = 4/group). **(A)** Muscle and **(B)** Lymph node **(C)** Liver, **(D)** Heart. Data plotted as mean ± SEM. Source data are provided as a Source Data file.

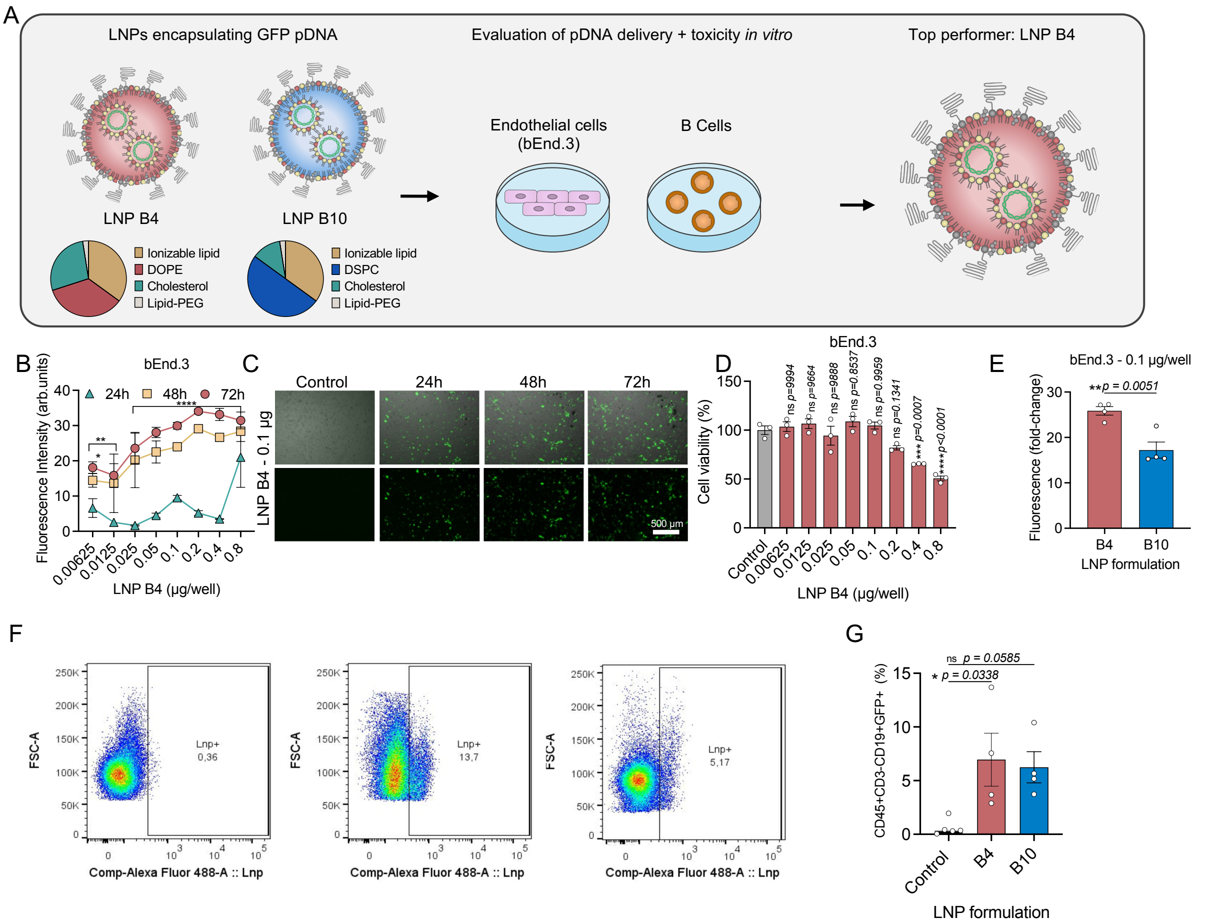

Supplementary Fig. 2: Top-performing LNPs induced enhanced in vitro GFP expression in endothelial and B cells. (A) Schematic of the 2 top-performing LNPs encapsulating DNA encoding GFP for in vitro screening of transfection efficiency in endothelial and B cells. **(B)** Quantification of GFP expression was measured after 24 h, 48 h, and 72h. **(C)** Representative GFP fluorescence **(Bottom)** and overlaid on brightfield **(Top)** photomicrographs after treatment of LNP B4 in endothelial cells (n = 4/group). **(D)** cell viability was measured after 72 h in endothelial cells transfected with LNP B4 at different doses (n = 3/group). **(E)** Quantification of GFP expression after treatment with B4 and B10 LNPs in endothelial cells. **(F)** Representative density plots illustrating the gating strategy for CD45+CD3-CD19+GFP+ in splenocytes treated with B4 or B10. **(G)** Quantification of GFP+ expression following treatment with B4 and B10 LNPs in B cells from mouse splenocytes. Data are presented as mean ± SEM (n = 4/group). **(B, F, D, G)** One-way ANOVA with Dunnet's post hoc test for 72h compared to control; ns not significant, *p<0.05, **p<0.01***p<0.001 ****p<0.0001. **(E, G)** Two-tailed unpaired t-test; *p<0.05, **p<0.01. Source data are provided as a Source Data file.

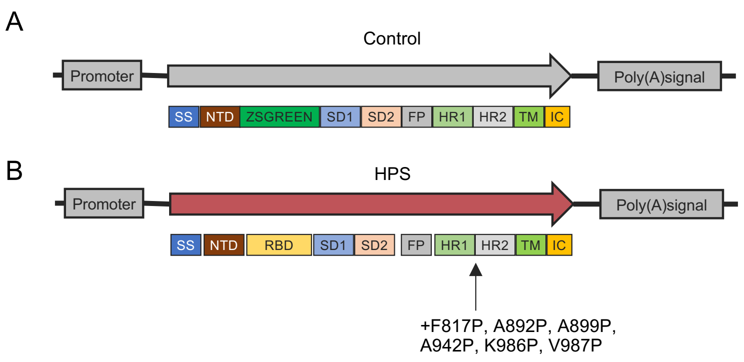

Supplementary Fig. 3: Plasmid DNAs (A) pZsGreen-N1 (Clontech laboratories, no. 632448) encoding ZsGreen, a green fluorescent protein (GFP) **(B)** recombinant HexaPro Spike plasmid (Addgene, no. 154754). Source data are provided as a Source Data file.

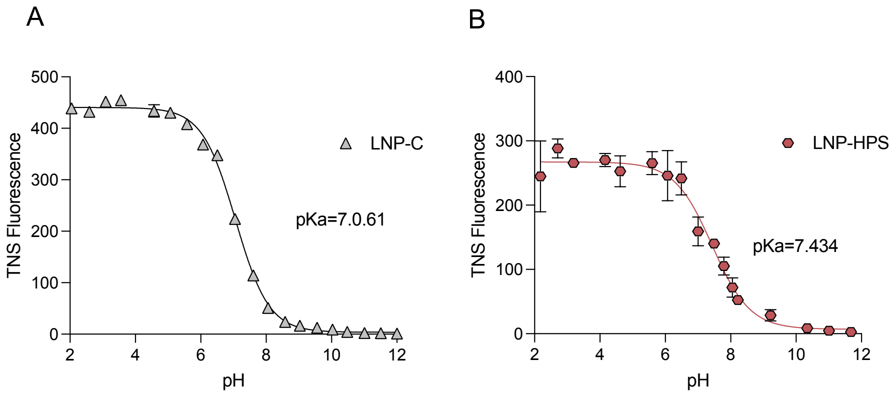

Supplementary Fig. 4: pKa measurements of LNP-C (A) and LNP-HPS (B) via TNS fluorescence assay. (*n* = 6/group). Data are presented as mean ± SEM. Source data are provided as a Source Data file.

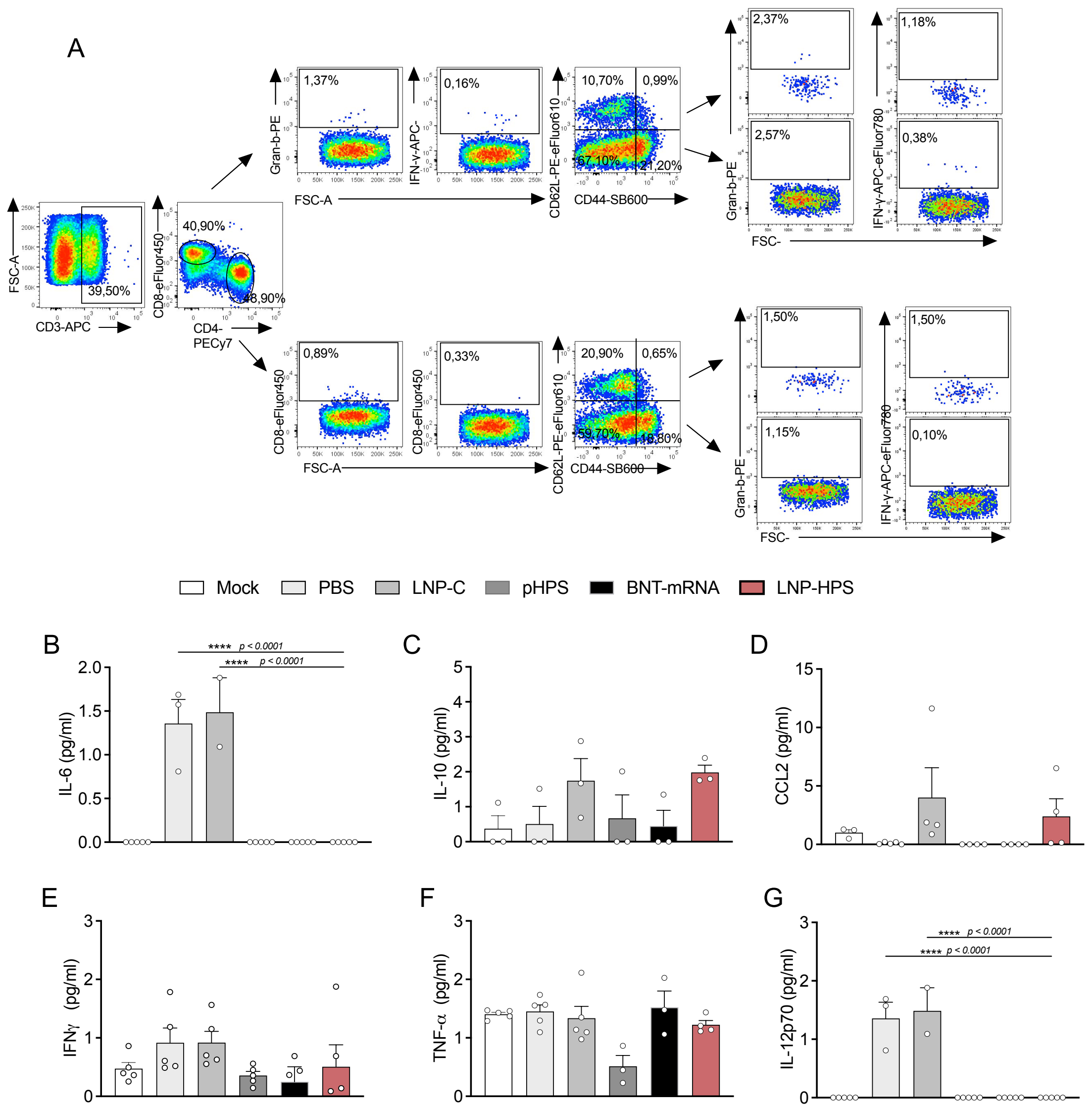

Supplementary Fig. 5: Gating strategy and serum cytokine and chemokines measurements. (A) Representative density plots showing the gating strategy. T cell subsets were gated on CD45+ live cells following the gate for single cells (FSC-H x FSC-A). CD3-positive cells were further subdivided into CD4 and CD8 T cells. Within each subset, memory subpopulations were assessed, including central memory (CD44+CD62L+), effector/effector memory (CD44+CD62L-), and naive cells (CD44-CD62L+). IFN-gamma and granzyme-b expression were evaluated in all subsets. **(B-G)** Serum cytokines and chemokines were quantified in K18-ACE-2 mice were immunized with controls and LNP-HPS and boosted with equivalent doses 3 weeks later. Immunized mice were challenged with lethal 6 x 10^4 PFU of SARS-CoV-2 variants Gamma lineage (P.1). Levels of cytokines in serum of mice were measured by at 5 days post-infection (n = 5/group). Data are presented as mean ± SEM. One-way ANOVA followed by Dunnett's multiple comparison test. ****p<0.0001. Source data are provided as a Source Data file.

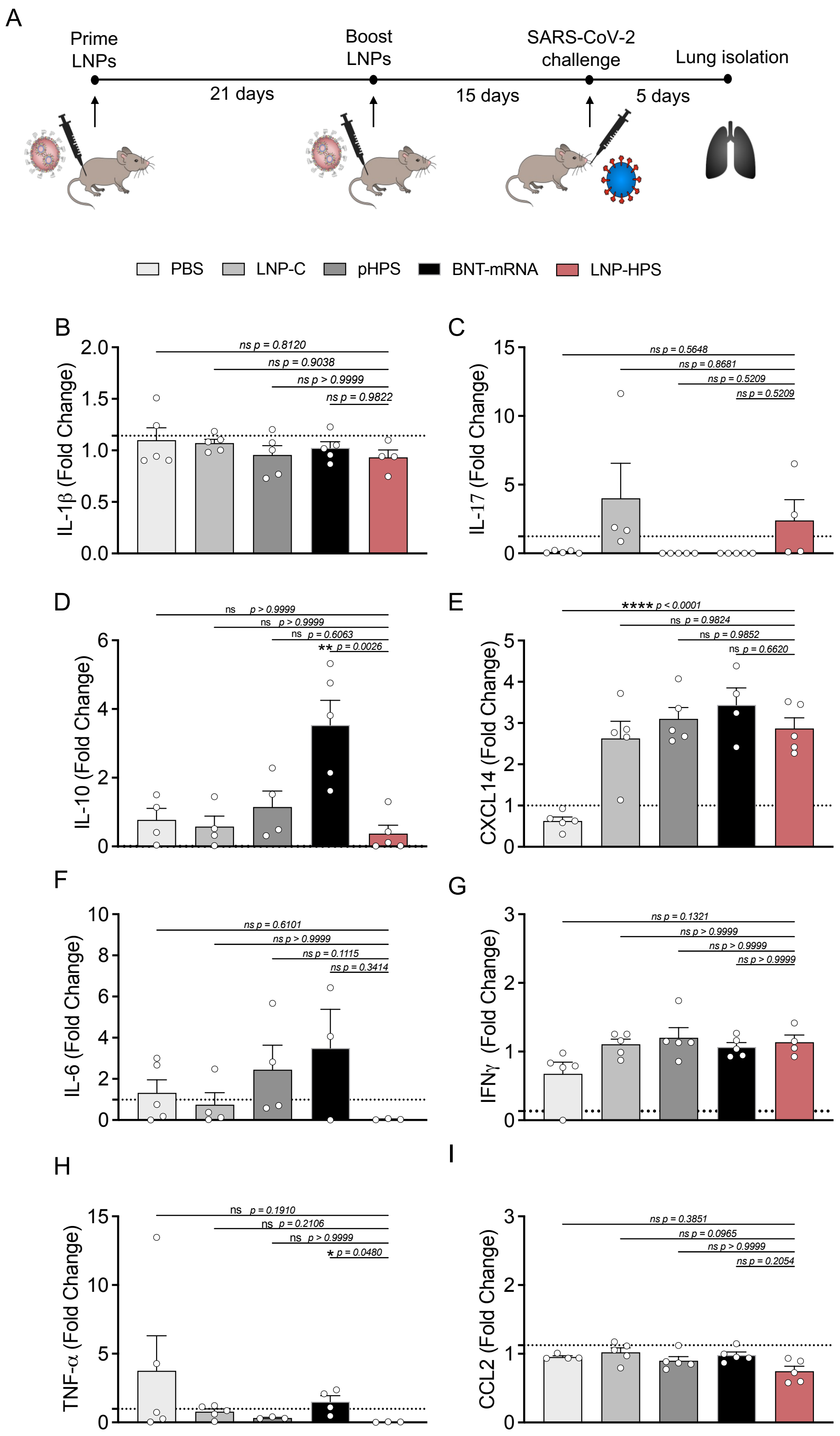

A
Prime LNPs
Boost LNPs
SARS-CoV-2 challenge
Lung isolation
21 days
15 days
5 days
PBS
LNP-C
pHPS
BNT-mRNA
LNP-HPS
B
IL-1β (Fold Change)
ns p = 0.8120
ns p = 0.9038
ns p > 0.9999
ns p = 0.9822
C
IL-17 (Fold Change)
ns p = 0.5648
ns p = 0.8681
ns p = 0.5209
ns p = 0.5209
D
IL-10 (Fold Change)
ns p > 0.9999
ns p > 0.9999
ns p = 0.6063
** p = 0.0026
E
CXCL14 (Fold Change)
**** p < 0.0001
ns p = 0.9824
ns p = 0.9852
ns p = 0.6620
F
IL-6 (Fold Change)
ns p = 0.6101
ns p > 0.9999
ns p = 0.1115
ns p = 0.3414
G
IFNγ (Fold Change)
ns p = 0.1321
ns p > 0.9999
ns p > 0.9999
ns p > 0.9999
H
TNF-α (Fold Change)
ns p = 0.1910
ns p = 0.2106
ns p > 0.9999
* p = 0.0480
I
CCL2 (Fold Change)
ns p = 0.3851
ns p = 0.0965
ns p > 0.9999
ns p = 0.2054

Supplementary Fig. 6: Levels of cytokines. (A) Scheme of immunization: K18-ACE-2 mice were immunized with controls and LNP-HPS, and boosted with equivalent doses 3 weeks later**.** Fold change in the gene expression of the indicated cytokines and chemokines **(B)** IL-1β, **(C)** IL-17, **(D)** IL-10, **(E)** CXCL14, **(F)** IL-6, **(G)** IFNγ, **(H)** TNF, and **(I)** CCL2, as determined by RT-qPCR, in lung homogenates of immunized and controls K18-hACE-2 mice infection (Mock $n = 4$; PBS, LNP-C, pHPS, BNT-mRNA, and LNP-HPS $n = 5$). HPRT was used as a reference gene**.** Data are presented as mean ± SEM. One-way ANOVA followed by Tukey's multiple comparison test. *$p<0.05$, **$p<0.01$, ****$p<0.0001$. Source data are provided as a Source Data file.

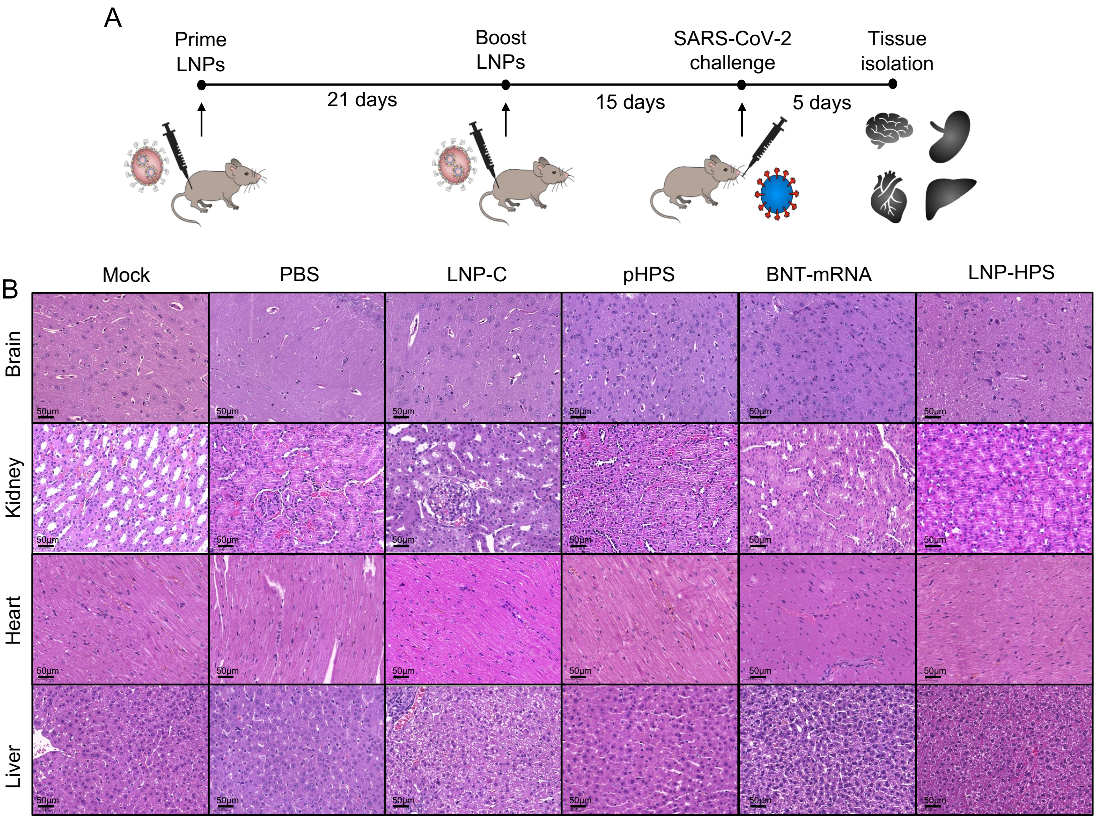

Supplementary Fig. 7: Histopathological analysis of P.1 SARS-CoV-2 infection in K18-hACE2 mice. (A) Scheme of immunization. K18-ACE-2 mice were immunized with controls and LNP-HPS and boosted with equivalent doses 3 weeks later. Immunized mice were challenged with lethal 6 x 10^4 PFU of SARS-CoV-2 variants Gamma lineage (P.1). Brain, kidney, heart and liver were harvested at 5 days post-infection for all immunized groups for histopathological analysis. **(B)** Histopathological analysis at 20 x magnification of brain, kidney, heart and liver at 5 days post-infection with the P.1 strain (n = 5/group), reveals no significant histological changes between the groups. Source data are provided as a Source Data file.

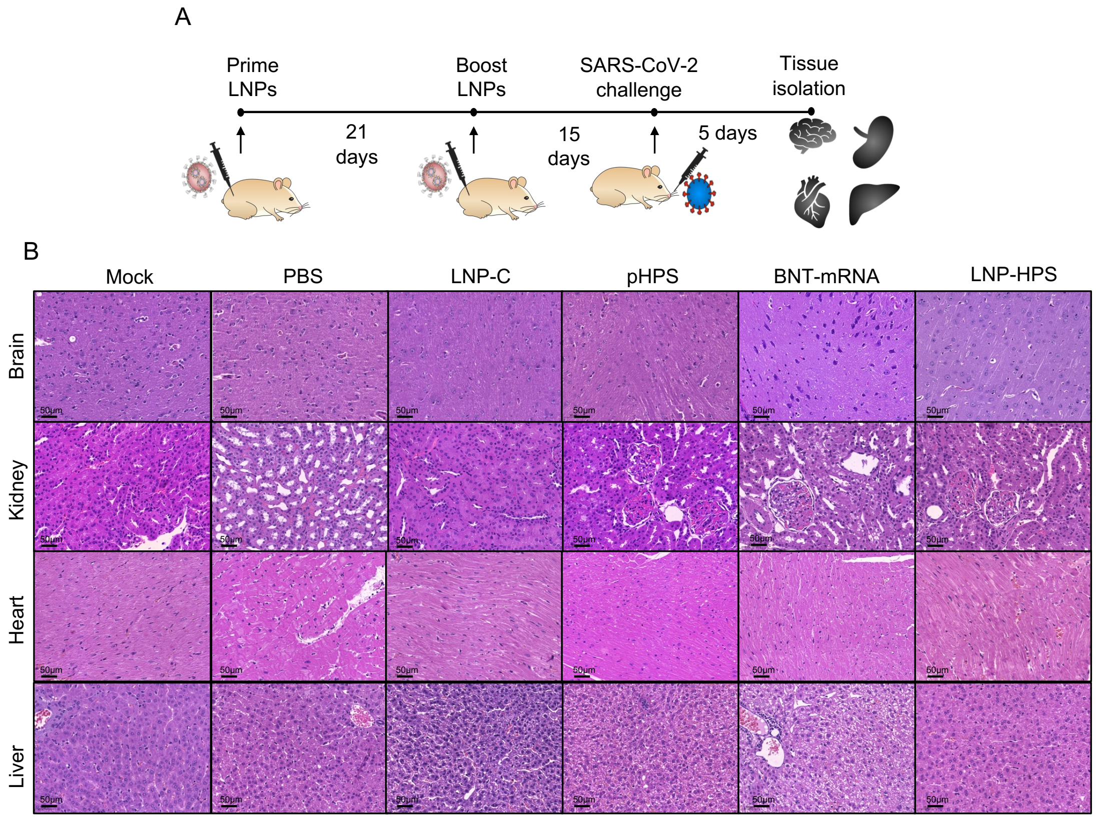

Supplementary Fig. 8: Histopathological analysis of P.1 SARS-CoV-2 infection in hamsters. **(A)** Scheme of immunization. Syrian hamsters were immunized with controls and LNP-HPS and boosted with equivalent doses 3 weeks later. Immunized hamsters were challenged with 6×10^5 PFU of SARS-CoV-2 variants Gamma lineage (P.1). Brain, kidney, heart and liver were harvested at 5 days post-infection for all immunized groups for histopathological analysis. **(B)** Histopathological analysis at 20 x magnification of brain, kidney, heart and liver at 5 days post-infection with the P.1 strain (*n* = 5/group), reveals no significant histological changes between the groups. Source data are provided as a Source Data file.

Supplementary Table 1: Composition of ionizable lipid nanoparticle formulations

LNP formulation	Ionizable lipid:b-DNA ratio	Molar ratio %				
		Ionizable lipids	Helper lipids	Cholesterol	Lipid-PEG	
B1	10:1	35	15	47,5	2,5	DOPE
B2	10:1	35	20	42,5	2,5	DOPE
B3	10:1	35	25	37,5	2,5	DOPE
B4	10:1	35	35	27,5	2,5	DOPE
B5	10:1	35	50	12,5	2,5	DOPE
B6	10:1	35	15	47,5	2,5	DSPC
B7	10:1	35	20	42,5	2,5	DSPC
B8	10:1	35	25	37,5	2,5	DSPC
B9	10:1	35	35	27,5	2,5	DSPC
B10	10:1	35	50	12,5	2,5	DSPC
B11	10:1	35	15	47,5	2,5	DOTAP
B12	10:1	35	20	42,5	2,5	DOTAP
B13	10:1	35	25	37,5	2,5	DOTAP
B14	10:1	35	35	27,5	2,5	DOTAP
B15	10:1	35	50	12,5	2,5	DOTAP

Abbreviations: LNP: ionizable lipid-nanoparticles; b-DNA: barcoded DNA; DOPE: 1,2-Dioleoyl-sn-glycero-3-phosphoethanolamine; DSPC:1,2-distearoyl-sn-glycero-3-phosphocholine; DOTAP:1,2-dioleoyl-3-trimethylammonium-propane.

Supplementary Table 2: Oligonucleotide sequences of b-DNA

LNP formulation	b-DNA added	Oligonucleotide sequences
B1	TAACGCACCT	A*G*A*CGTGTGCTCTTCCGATCT TAACGCACCT NNNNNNNNNN AGATCGGAAGAGCGTCG*T*G*T
B2	GAGGGTACTT	A*G*A*CGTGTGCTCTTCCGATCT GAGGGTACTT NNNNNNNNNN AGATCGGAAGAGCGTCG*T*G*T
B3	TGTCTCCCAT	A*G*A*CGTGTGCTCTTCCGATCT TGTCTCCCAT NNNNNNNNNN AGATCGGAAGAGCGTCG*T*G*T
B4	GGAGAAACAG	A*G*A*CGTGTGCTCTTCCGATCT GGAGAAACAG NNNNNNNNNN AGATCGGAAGAGCGTCG*T*G*T
B5	ATGATCGTCG	A*G*A*CGTGTGCTCTTCCGATCT ATGATCGTCG NNNNNNNNNN AGATCGGAAGAGCGTCG*T*G*T
B6	TTGCAGCCTT	A*G*A*CGTGTGCTCTTCCGATCT TTGCAGCCTT NNNNNNNNNN AGATCGGAAGAGCGTCG*T*G*T
B7	CGTACAAACG	A*G*A*CGTGTGCTCTTCCGATCT CGTACAAACG NNNNNNNNNN AGATCGGAAGAGCGTCG*T*G*T
B8	ATCCATGAGG	A*G*A*CGTGTGCTCTTCCGATCT ATCCATGAGG NNNNNNNNNN AGATCGGAAGAGCGTCG*T*G*T
B9	TTCCACGATG	A*G*A*CGTGTGCTCTTCCGATCT TTCCACGATG NNNNNNNNNN AGATCGGAAGAGCGTCG*T*G*T
B10	GAATGCTGAC	A*G*A*CGTGTGCTCTTCCGATCT GAATGCTGAC NNNNNNNNNN AGATCGGAAGAGCGTCG*T*G*T
B11	TCTCGCCTTT	A*G*A*CGTGTGCTCTTCCGATCT TCTCGCCTTT NNNNNNNNNN AGATCGGAAGAGCGTCG*T*G*T
B12	GCTGGGAATT	A*G*A*CGTGTGCTCTTCCGATCT GCTGGGAATT NNNNNNNNNN AGATCGGAAGAGCGTCG*T*G*T
B13	GACACGTTCT	A*G*A*CGTGTGCTCTTCCGATCT GACACGTTCT NNNNNNNNNN AGATCGGAAGAGCGTCG*T*G*T
B14	TTCGCATCTG	A*G*A*CGTGTGCTCTTCCGATCT TTCGCATCTG NNNNNNNNNN AGATCGGAAGAGCGTCG*T*G*T
B15	CAGATCAGAG	A*G*A*CGTGTGCTCTTCCGATCT CAGATCAGAG NNNNNNNNNN AGATCGGAAGAGCGTCG*T*G*T

Abbreviations: LNP: ionizable lipid-nanoparticles; b-DNA: barcoded DNA.

Supplementary Table 3: Characterization of b-DNA encapsulated LNPs

LNP formulation	Hydrodynamic diameter (nm)	Polydispersity index (PDI)	Zeta Potential (mV)	
**B1**	106 ± 2	0.114 ± 0.009	-9.37 ± 9.15	**DOPE**
**B2**	117 ± 5	0.187 ± 0.017	-6.14 ± 6.02	
**B3**	111 ± 4	0.181 ± 0.015	-2.2 ± 6.64	
**B4**	115 ± 3	0.138 ± 0.030	-1.41 ± 7.26	
**B5**	127 ± 2	0.141 ± 0.002	-8.9 ± 8.75	
**B6**	108 ± 3	0.242 ± 0.008	-10.05 ± 7.85	**DSPC**
**B7**	96 ± 8	0.221 ± 0.011	-12.35 ± 6.85	
**B8**	171 ± 1	0.229 ± 0.009	-9.67 ± 11.2	
**B9**	233 ± 6	0.224 ± 0.018	-5.31 ± 5.41	
**B10**	183 ± 10	0.235 ± 0.020	1.46 ± 5.08	
**B11**	118 ± 1	0.200 ± 0.002	-7.84 ± 6.97	**DOTAP**
**B12**	115 ± 3	0.124 ± 0.011	-23.03 ± 7.61	
**B13**	110 ± 1	0.102 ± 0.021	27.53 ± 4.41	
**B14**	162 ± 11	0.253 ± 0.016	16.8 ± 9.55	
**B15**	84 ± 5	0.261 ± 0.038	20.37 ± 6.01	

Abbreviations: b-DNA: barcoded DNA; LNP: ionizable lipid-nanoparticles; nm: nanometer; mV: millivolts; PDI: polydispersity index; DOPE: 1.2-Dioleoyl-sn-glycero-3-phosphoethanolamine; DSPC: 1.2-distearoyl-sn-glycero-3-phosphocholine; DOTAP: 1.2-dioleoyl-3-trimethylammonium-propane.
