# Supplementary material for: Nanoparticle-based DNA vaccine protects against SARS-CoV-2 variants in female preclinical models
Source: Nat Commun. 2024 Jan 18;15:590. doi: 10.1038/s41467-024-44830-1 (PMC10796936; doi:10.1038/s41467-024-44830-1)
Supplement: Supplementary file 3 — Reporting Summary [file 41467_2024_44830_MOESM3_ESM.pdf]

nature portfolio

Corresponding author(s): Pedro Pires Goulart Guimaraes

Last updated by author(s): Nov 19, 2023

Reporting Summary

Nature Portfolio wishes to improve the reproducibility of the work that we publish. This form provides structure for consistency and transparency in reporting. For further information on Nature Portfolio policies, see our Editorial Policies and the Editorial Policy Checklist.

Statistics

For all statistical analyses, confirm that the following items are present in the figure legend, table legend, main text, or Methods section.

n/a	Confirmed	
☐	☒	The exact sample size (*n*) for each experimental group/condition, given as a discrete number and unit of measurement
☐	☒	A statement on whether measurements were taken from distinct samples or whether the same sample was measured repeatedly
☐	☒	The statistical test(s) used AND whether they are one- or two-sided<br>*Only common tests should be described solely by name; describe more complex techniques in the Methods section.*
☒	☐	A description of all covariates tested
☒	☐	A description of any assumptions or corrections, such as tests of normality and adjustment for multiple comparisons
☐	☒	A full description of the statistical parameters including central tendency (e.g. means) or other basic estimates (e.g. regression coefficient) AND variation (e.g. standard deviation) or associated estimates of uncertainty (e.g. confidence intervals)
☐	☒	For null hypothesis testing, the test statistic (e.g. *F*, *t*, *r*) with confidence intervals, effect sizes, degrees of freedom and *P* value noted<br>*Give P values as exact values whenever suitable.*
☒	☐	For Bayesian analysis, information on the choice of priors and Markov chain Monte Carlo settings
☒	☐	For hierarchical and complex designs, identification of the appropriate level for tests and full reporting of outcomes
☒	☐	Estimates of effect sizes (e.g. Cohen's *d*, Pearson's *r*), indicating how they were calculated

Our web collection on statistics for biologists contains articles on many of the points above.

Software and code

Policy information about availability of computer code

Data collection	Excel from Office 365 for windows 10
Data analysis	PrismGrahPad 10.0, GraphPad Prism V8.0 (GraphPad software), ZEN Microscopy Software 2.1 (blue edition), Python 3.10 and FlowJo V10.8.1 (BD).

For manuscripts utilizing custom algorithms or software that are central to the research but not yet described in published literature, software must be made available to editors and reviewers. We strongly encourage code deposition in a community repository (e.g. GitHub). See the Nature Portfolio guidelines for submitting code & software for further information.

Data

Policy information about availability of data

All manuscripts must include a data availability statement. This statement should provide the following information, where applicable:

- Accession codes, unique identifiers, or web links for publicly available datasets
- A description of any restrictions on data availability
- For clinical datasets or third party data, please ensure that the statement adheres to our policy

All data are available in the main text or supplementary materials. Source data are provided with this paper. Codes used or generated during the current study are available upon request from the corresponding author. Due to privacy and ethical considerations, code sharing is restricted, but interested parties may contact corresponding author to request access to the code.

Research involving human participants, their data, or biological material

Policy information about studies with human participants or human data. See also policy information about sex, gender (identity/presentation), and sexual orientation and race, ethnicity and racism.

Reporting on sex and gender	n/a
Reporting on race, ethnicity, or other socially relevant groupings	n/a
Population characteristics	n/a
Recruitment	n/a
Ethics oversight	n/a

Note that full information on the approval of the study protocol must also be provided in the manuscript.

Field-specific reporting

Please select the one below that is the best fit for your research. If you are not sure, read the appropriate sections before making your selection.

☒ Life sciences ☐ Behavioural & social sciences ☐ Ecological, evolutionary & environmental sciences

For a reference copy of the document with all sections, see nature.com/documents/nr-reporting-summary-flat.pdf

Life sciences study design

All studies must disclose on these points even when the disclosure is negative.

Sample size	The sample size was calculated to satisfy the minimum requirements for conducting statistical analysis. The sample sizes were determined by allowable error size, accuracy, resouces, and need for statistical analysis (generally n>=3 throughout all the studies). The sample sizes are clearly described in each figure legend. Experiments were conducted independently at least three times, with each assay including a minimum of two technical replicates.
Data exclusions	No data were excluded
Replication	The experiments were repeated independently at three times, if the statistical significance was P<0.05. All attempts at replication were successful.
Randomization	Animals were randomized using the random sequence generator from https://www.random.org. For other experiments, all samples were randomly allocated into experimental groups, as there was no covariate in the study design.
Blinding	All the investigators were blinded to group allocation during data collection and analysis.

Reporting for specific materials, systems and methods

We require information from authors about some types of materials, experimental systems and methods used in many studies. Here, indicate whether each material, system or method listed is relevant to your study. If you are not sure if a list item applies to your research, read the appropriate section before selecting a response.

Materials & experimental systems

n/a	Involved in the study	
☐	☒	Antibodies
☐	☒	Eukaryotic cell lines
☒	☐	Palaeontology and archaeology
☐	☒	Animals and other organisms
☒	☐	Clinical data
☒	☐	Dual use research of concern
☒	☐	Plants

Methods

n/a	Involved in the study	
☒	☐	ChIP-seq
☐	☒	Flow cytometry
☒	☐	MRI-based neuroimaging

Antibodies

Antibodies used	-anti-mouse CD45 Pacific Orange(REF MCD4540 LOT 2388876, 30-F11, ThermoFisher)<br>-anti- mouse CD3 Pacific Orange(REF 17-0032-82 LOT 2400617, 30-F11, ThermoFisher)<br>-anti- mouse CD4 PE-Cy7, (REF 25-0041-82 LOT 2450690, GK1.5, ThermoFisher)<br>-anti- mouse CD8 eFluor 450, (REF 48-0081-82 LOT 2527379, 53-6.7, ThermoFisher)<br>-anti- mouse CD44 SB600, (REF 63-0441-082 LOT 434571A, IM7, ThermoFisher)<br>-anti- mouse CD62L PE-eFluor610, (REF 61-0621-82 LOT 23055625, MEL-14, ThermoFisher)<br>-anti- mouse IFN-ɣ APC-eFluor 780, (REF 47-7311-82 LOT 2518473, XMG1.2, ThermoFisher)<br>-anti- mouse Gran-b PE, (REF 128898-82 LOT 2633426, NGZB, ThermoFisher)<br>-Biotin-mouse anti American and Syrian Hamster IgG (REF 554010 LOT 65198, BD Biosciences)<br>-Avidin-Horseradish Peroxidase (HRP) (REF 554058 LOT 89268, BD Biosciences)<br>-Anti-mouse IgG – Peroxidase (REF A4416 LOT MFCD00162644, Sigma-Aldrich)<br>-Anti-mouse IgA – Peroxidase (REF 1040-05 LOTC0919-N930B, SouthernBiotech)<br>-IgG goat-rabbit , Alexa Flúor 647 (REF A21245 LOT 1729791 SinoBiological)<br>-SARS-CoV-2 (2019-nCoV) Spike RBD Antibody, Rabbit PAb, Antigen Affinity Purified (Cat 40592-T62)
Validation	All antibodies were verified by the supplier and each lot has been quality tested. All the antibodies used are from commercial sources and have been validated by the vendors. Validation statements (species, application et al.) and data are available on the manufacture's website.

Eukaryotic cell lines

Policy information about cell lines and Sex and Gender in Research

Cell line source(s)	RAW 264.7, L929, bEnd.3, C2C12, HEK-293, JAWSII, Vero CCL-81 cells. RAW 264.7, JAWSII and Vero CCL-81 were purchased from Rio de Janeiro's cell bank: https://bcrj.org.br/. L929, bEnd.3 and HEK-293 were purchased from ATCC
Authentication	We did not conduct explicit authentication procedures on the cellular systems utilized.
Mycoplasma contamination	Cells were periodically monitored as negative for mycoplasma using PCR-based method
Commonly misidentified lines<br>(See ICLAC register)	No Commonly misidentified lines were used according to the version 12, release 16 Jan 2023, on ICLAC website (https://iclac.org/databases/cross-contaminations/)

Animals and other research organisms

Policy information about studies involving animals; ARRIVE guidelines recommended for reporting animal research, and Sex and Gender in Research

Laboratory animals	This study received approval from the Ethical Committees on the use of animals in research at the Federal University of Minas Gerais (UFMG). The experiments involving mice and hamsters adhered to institutional guidelines for animal ethics and were approved by the Institutional Ethics Committees at UFMG, specifically Commission on Animal Use (CEUA) 177/2020, 245/2021, and 165/2021, for C57BL/6 mice, K18-hACE2 transgenic mice, and Syrian hamsters, respectively. Female C57BL/6 mice and female Syrian hamsters were purchased from Biotério Central at UFMG. Human Angiotensin Converting Enzyme transgenic mice (K18-hACE2) in the C57BL/6 background, originally from Jackson Laboratories, 8–10 weeks old, were bred at UFMG animal facilities and only female K18-hACE2 mice were utilized as a model of severe COVID-19. Female Syrian Hamsters, 8–10 weeks old, served as a model of mild COVID-19. The experiments were conducted in accordance with the recommendations in the Guide for the Care and Use of Laboratory Animals of the Brazilian National Council of Animal Experimentation (CONCEA). Infections of K18-ACE2 and Syrian hamsters were performed in the Animal Biosafety Level 3 (ABSL-3) facility at the Institute of Biological Sciences from UFMG. All animals were maintained with 12h light/dark cycle with humidity of 50-58% and temperature of 25ºC. The SARS-CoV-2 viral strain used in this study belonged to the lineage P.1 (EPI_ISL_13017802) and Omicron (EPI_ISL_7699344) variants. Viral stocks were propagated in Vero CCL81 in a humidified incubator at 37 °C with 5% CO2 and monitored for cytopathic effects (CPE) daily up to 72 h. Viruses were titrated in Vero CCL81 cells by plaque forming units (PFU) assay, and viral aliquots were kept at −80 °C until further use.
Wild animals	No wild animals were used in the study.
Reporting on sex	Exclusively female mice were employed in this study as a precaution against the elevated aggression levels observed in male mice. This choice is made to mitigate the increased biosafety risk in the BSL3 environment during high-titer intranasal challenges with virulent SARS-CoV-2. Additionally, females are slightly more susceptible to develop respiratory disease resembling severe COVID-19 under the experimental conditions(1).<br><br>(1) Yinda, C. K. et al. K18-hACE2 mice develop respiratory disease resembling severe COVID-19. PLoS Pathog 17, e1009195 (2021).
Field-collected samples	No field collected samples were used in the study.
Ethics oversight	The treatment protocol was previously approved by the Institutional Animal Care and Use Committee at UFMG for C57BL/6 mice (177/2020), K18-hACE2 transgenic mice (245/2021) and Syrian hamsters (165/2021).

Note that full information on the approval of the study protocol must also be provided in the manuscript.

Plants

Seed stocks: n/a

Novel plant genotypes: n/a

Authentication: n/a

Flow Cytometry

Plots

Confirm that:

☒ The axis labels state the marker and fluorochrome used (e.g. CD4-FITC).

☒ The axis scales are clearly visible. Include numbers along axes only for bottom left plot of group (a 'group' is an analysis of identical markers).

☒ All plots are contour plots with outliers or pseudocolor plots.

☒ A numerical value for number of cells or percentage (with statistics) is provided.

Methodology

Sample preparation	Sample preparation to assess the T cell response, spleens from immunized mice were harvested 5 days post-infection and macerated using a 70µm pore cell strainer (Cell Strainer, BD Falcon) followed by erythrocyte lysis. Splenocytes were plated and stimulated overnight with 10µg/mL of RBD S-1 Spike protein. Phorbol 12-myristate 13-acetate (PMA) plus ionomycin (1 µg/mL) (Sigma, 25 ng/mL) was used as positive control. Next, stimulated splenocytes were cultivated for 4 h at 37 °C in RPMI supplemented with 10% FBS, 2 mM L-glutamine, 50 µg/mL streptomycin, and 50 units/mL penicillin, in the presence of Brefeldin A (ThermoFisher).
Instrument	BD LSRFortessa
Software	Collection of data was performed using Digital DIVA hardware ans software analysis using FlowJo V10.8.1 (BD)
Cell population abundance	No sorting was realyzed
Gating strategy	T cell subsets were gated on CD45+ live cells after the gate of single cells (FSC-H x FSC-A). CD3 positive were subdivided into CD4 and CD8 T cells. In aach subset was evaluate memory subpopulations, central memory (CD44+CD62L+), effector/effector memory (CD44+CD62L-) and naive cells (CD44-CD62L+). In all subsets were evaluated IFN-gamma and granzyme-b. GFP expression in B cells (CD3-CD19+) was also evaluated.

☒ Tick this box to confirm that a figure exemplifying the gating strategy is provided in the Supplementary Information.
